# Exploring how AI adoption in the workplace affects employees: a bibliometric and systematic review

**DOI:** 10.3389/frai.2024.1473872

**Published:** 2024-11-14

**Authors:** Malika Soulami, Saad Benchekroun, Asiya Galiulina

**Affiliations:** ^1^Formerly at Laboratory of Studies and Research in Management of Organizations and Territories (ERMOT), Faculty of Legal, Economic, and Social Sciences of Fez, Sidi Mohamed Ben Abdellah University, Fez, Morocco; ^2^Laboratory of Research in Economy and Management of Organisations (LAREMO), National School of Business and Management of Beni Mellal, Sultan Moulay Slimane University, Beni Mellal, Morocco

**Keywords:** artificial intelligence, bibliometric review, employee well-being, systematic review, workplace stress

## Abstract

**Introduction:**

The adoption of artificial intelligence (AI) in the workplace is changing the way organizations function, and profoundly affecting employees. These organizational changes raise crucial questions about the employee’s future and well-being. Our study aims to explore the intersection between artificial intelligence and employee well-being through a bibliometric review and a contextual analysis.

**Methodology:**

Carried out in May 2024, our study is divided into two phases. The first phase, dedicated to bibliometric review, was conducted using the PRISMA method, and explored the Scopus and Web of Science databases for the period from 2015 to 2024. A total of 92 articles were selected for quantitative analysis using VOSviewer software. The second phase is based on an in-depth systematic analysis of 25 articles selected from those previously identified. These articles were selected on the basis of their relevance to the research question, and were subjected to in-depth thematic analysis using NVivo software.

**Results:**

The bibliometric analysis results reveal a significant increase in publications starting from the year 2020, highlighting advancements in research, primarily in the United States and China. The co-occurrence analysis identifies four main clusters: ethics, work autonomy, employee stress, and mental health, thus illustrating the dynamics created by artificial intelligence in the professional environment. Furthermore, the systematic analysis has brought to light theoretical gaps and under-explored areas, such as the need to conduct empirical studies in non-Western cultural contexts and among diverse target groups, including older adults, individuals of different sexes, people with low education levels, and participants from various sectors, including primary and secondary industries, small manufacturing businesses, call centers, as well as public and private healthcare sectors.

**Conclusion:**

Existing literature emphasize the importance for organizations to implement supportive strategies aimed at mitigating the potential adverse effects of AI on employee well-being, while also leveraging its benefits to enhance workplace autonomy and satisfaction and promote AI-enabled innovation through employee creativity and self-efficacy.

## 1 Introduction

Employee well-being is a broad and multidisciplinary concept that encompasses both tangible aspects such as income and social benefits, and intangible aspects related to a sense of belonging, satisfaction, and motivation (Rath and Harter, 2010). These elements significantly influence not only the overall performance of employees but also their personal, family and social fulfillment (Chari et al., 2018). As with any societal change driven by technology, employee well-being is inevitably affected (Nazareno and Schiff, 2021). The shift toward artificial intelligence (AI) is reshaping not just the future of businesses but also the work environments and experiences of employees. The automation of tasks, replacement of the workforce, disappearance of certain jobs, reallocation of skills, and the need to acquire new competencies pose substantial challenges in today’s workplace (Didem and Anke, 2021; Nazareno and Schiff, 2021; Peters, 2017). The impact of AI on professional settings is complex and varied; while it allows some employees to focus on strategic and reflective tasks, freeing them from monotonous and repetitive activities, others view these changes with concern, fearing job loss and instability (Hagel et al., 2018; Stamate et al., 2021).In summary, while AI can enhance employee well-being by improving job satisfaction, overall health, and reducing stress levels, it also presents significant challenges that can adversely affect the workforce. This article explores the dynamic interconnection between artificial intelligence (AI) and employee well-being through a combined methodological approach that includes a bibliometric review and a systematic review. Utilizing data from the Scopus and Web of Science databases, we have selected 92 articles for the bibliometric analysis to identify current trends, recognize influential authors, and outline the main research axes. To enhance this analysis, we conducted a systematic review based on specific inclusion criteria outlined in our methodology section, selecting 25 articles that further our understanding of the applied theories, identify existing theoretical gaps, and highlight potential directions for future research. Our investigation is guided by several crucial questions:

- What is the publication trend of AI & Employee well-being?
- Which countries have contributed to AI & Employee well-being?
- Who are the top-cited authors in the field of AI & Employee well-being?
- Which journals are leading in the field of AI & Employee well-being?
- What are the most used keywords in AI & Employee well-being?
- What are the major theoretical gaps identified in the literature on AI & Employee well-being?
- What future research directions are suggested in the field of AI & Employee well-being?
- What theories have been most frequently utilized to study AI & Employee well-being?

The findings from this study are set to serve as a foundational for future researchers interested in the multifaceted role of AI in professional settings. Additionally, the insights provided will empower decision-makers, especially human resource managers, with a deeper understanding of AI’s dimensions. This knowledge will enable them to use AI not as a disruptor but as a tool to enhance employee performance and well-being.

In the following sections, the article will detail the methodology used for gathering and analyzing data. Subsequently, we will discuss the results of both the bibliometric and content analyses. Finally, the discussion will synthesize these findings, interpreting their implications for both theory and practice, and conclude with a summary of the research contributions and recommendations for future studies.

## 2 Methods

**Figure 1:**
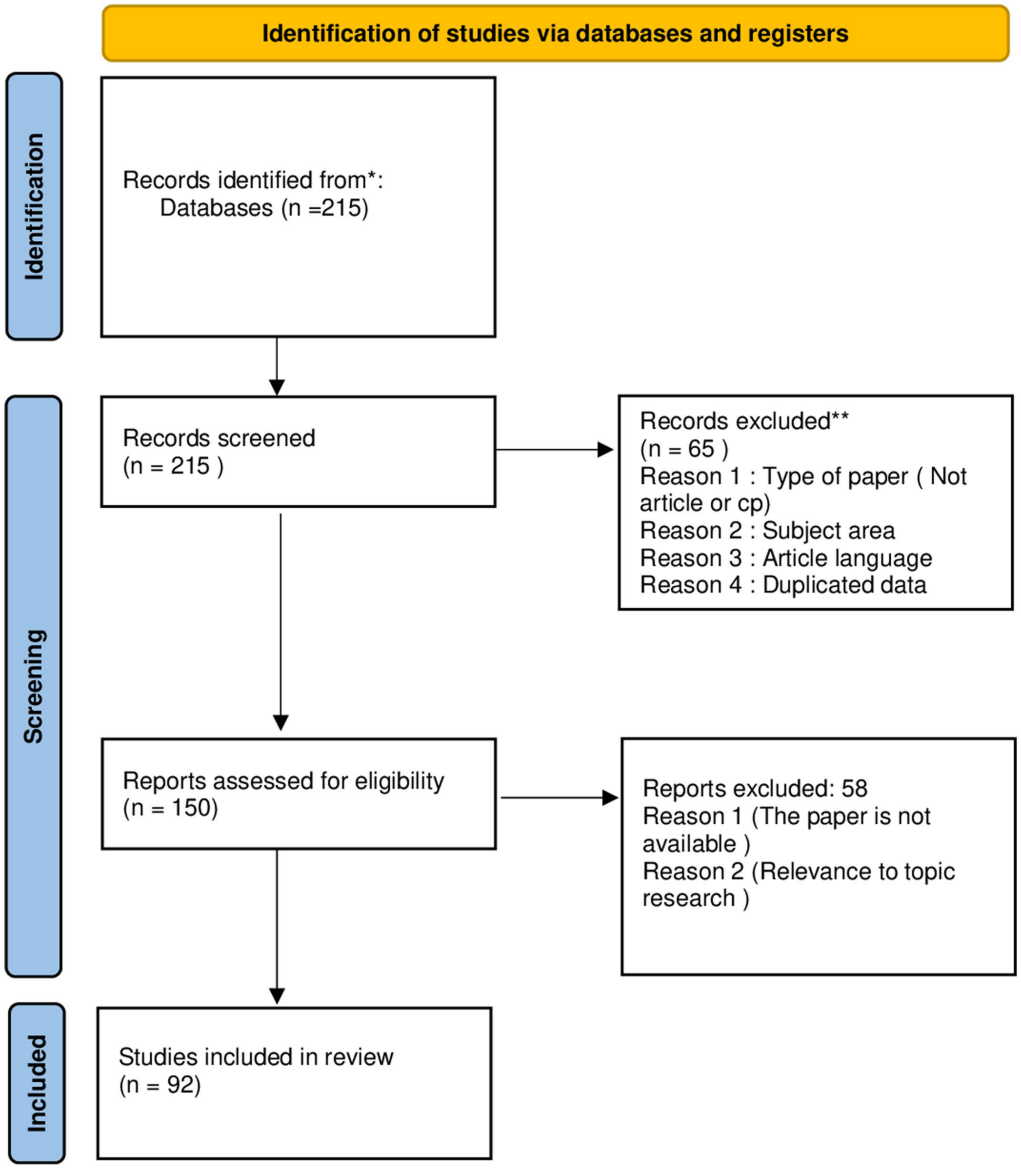
Research steps. The prisma website as a supporting source for the preparation of the diagram. https://www.prisma-statement.org/prisma-2020-flow-diagram.

To address our research questions, we adopted a method combining both a bibliometric review and a systematic review. The bibliometric review enabled a quantitative analysis of the data, focusing particularly on the number of publications per year, the most influential authors, the most relevant journals, and a co-occurrence analysis of keywords. In complement, the systematic review provided an in-depth analysis of the selected articles, allowing us to identify theoretical gaps, suggest future research perspectives, and examine the methodologies employed in the different studies. The data used for this analysis were extracted in May 2024 from Scopus and Web of Science, which are widely recognized for their comprehensive coverage and reliability (Chadegani et al., 2013). To ensure the rigor of the article selection process, we followed the PRISMA method, known for its capacity to enhance transparency and the rigor needed to conduct a literature review (Liberati et al., 2009) (see Figure 1).

The search query was formulated as follows: (“artificial intelligence” OR “AI”) AND (“employee well-being” OR “employee health” OR “workplace stress” OR “employee satisfaction”) AND (LIMIT TO (DOCTYPE, “ar”)) OR LIMIT-TO (DOCTYPE, “cp”)) AND (LIMIT-TO (LANGUAGE, “English”). This search query allowed us to select 92 articles, which were subsequently subjected to quantitative analysis using VOSviewer (version 1.6.20). VOSviewer is particularly suited for processing bibliographic data and creating graphical representations that help visualize the relationships between the studied concepts (Eck and Waltman, 2009). In the context of our analysis, we used this software to study the evolution of publications over time, identify the most influential authors, analyze the distribution of publications by journal, and perform a co-occurrence analysis of keywords. After importing the bibliographic data in RIS format into VOSviewer, we set the keyword frequency threshold to three occurrences, meaning that only keywords appearing at least three times in the articles were included in the analysis. A data-cleaning process was then carried out to remove redundant or similar keywords that could skew the results, such as “human” and “humans,” or “AI” and “artificial intelligence.” Similarly, overly similar terms like “worker” and “employee” were harmonized to ensure consistency in the analyzed data. The results from this analysis will be discussed in detail in the results section of this study.

Regarding the systematic review, we conducted a thorough and detailed qualitative analysis of the articles related to our research domain. From the 92 articles obtained from the bibliometric review, we refined the selection to retain only 25 articles. The articles selected for our systematic review meet strict inclusion criteria, requiring the presence of terms ‘Artificial Intelligence’ or equivalent expressions, ‘Employee Well-being’, ‘Job Satisfaction’, or similar expressions in the title, abstract, or keywords. Additionally, exclusion criteria were applied to eliminate articles that did not directly address artificial intelligence or aspects related to well-being, satisfaction, or health of employees. To strengthen our selection process, minimize research errors, and ensure a rigorous analysis, the three authors collaborated closely to evaluate each article. The analysis of the articles was carried out using the Nvivo14 software, which allowed us to organize and qualitatively analyze the data. The analysis process followed a five-step protocol, which will be detailed in the “Results” section. First, we generated a word cloud to visualize the most frequently used terms. Next, we created a word tree to explore the hierarchical relationships between the key concepts in the articles. Third, we examined the methodology adopted by each study. Fourth, we focused our analysis on the theories mobilized in the studies. Out of the 25 selected articles, we retained 15 for this step, excluding 10 articles that were limited to literature reviews. Finally, to create a hierarchical diagram in Nvivo, we imported the full text of the 25 articles into the software. During the reading process, we created several nodes (or codes) representing the main axes of research perspectives, such as “exploration of new research questions,” “adoption of alternative methodological approaches,” or “conducting empirical studies in other sectors.” Each node was then subdivided into sub-nodes, where we encoded the relevant parts of the text from each article.

## 3 Results

In alignment with the research steps previously outlined, this section is divided into two subsections. The first subsection discusses the findings of the bibliometric analysis, covering aspects such as the trends in publication years, the most influential authors, key journals, and clusters for keyword co-occurrence. The second subsection delves into the content analysis results, presenting elements such as word clouds, word trees, the methodologies employed by researchers, the theories utilized, the sectors examined, the themes explored, and the principal findings. This subsection also highlights the theoretical gaps identified and outlines future research directions.

### 3.1 Bibliometric review

#### 3.1.1 Annual publication

**Figure 2:**
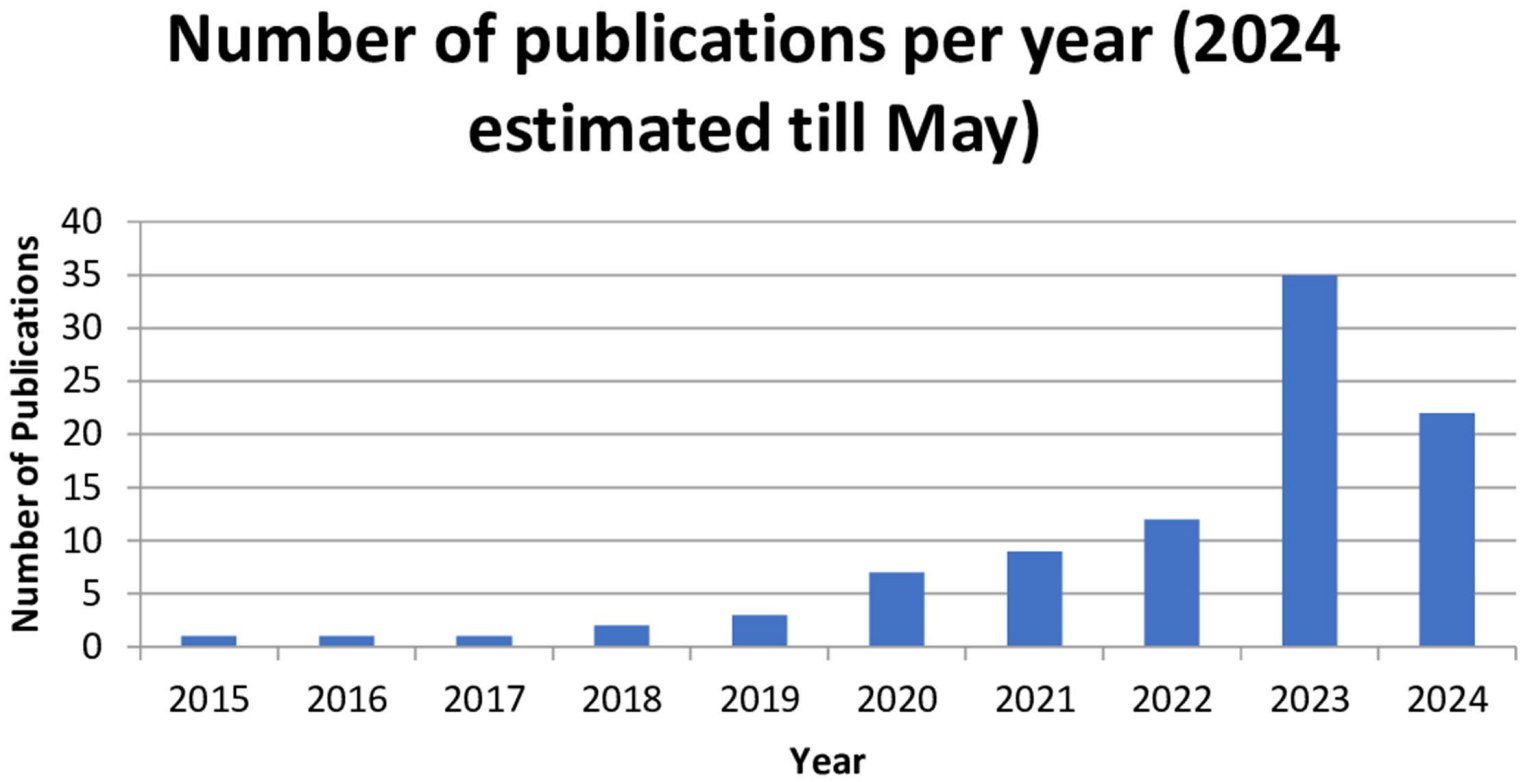
Annual publications.

This bar chart (Figure 2) illustrates the number of publications per year from 2015 to 2024, showing a clear increase in activity over time. Initially, the number of publications was relatively low between 2015 and 2018, but it began to rise significantly in 2020. This increase can be attributed to the COVID-19 pandemic (Kniffin et al., 2021), which caused substantial changes in the way businesses operate and had a profound impact on employee well-being. At the same time, the Fourth Industrial Revolution, characterized by the growing adoption of artificial intelligence, led to significant transformations in employment structures and work practices, redefining the future of work (Stamate et al., 2021).

#### 3.1.2 Publications per authors

**Figure 3:**
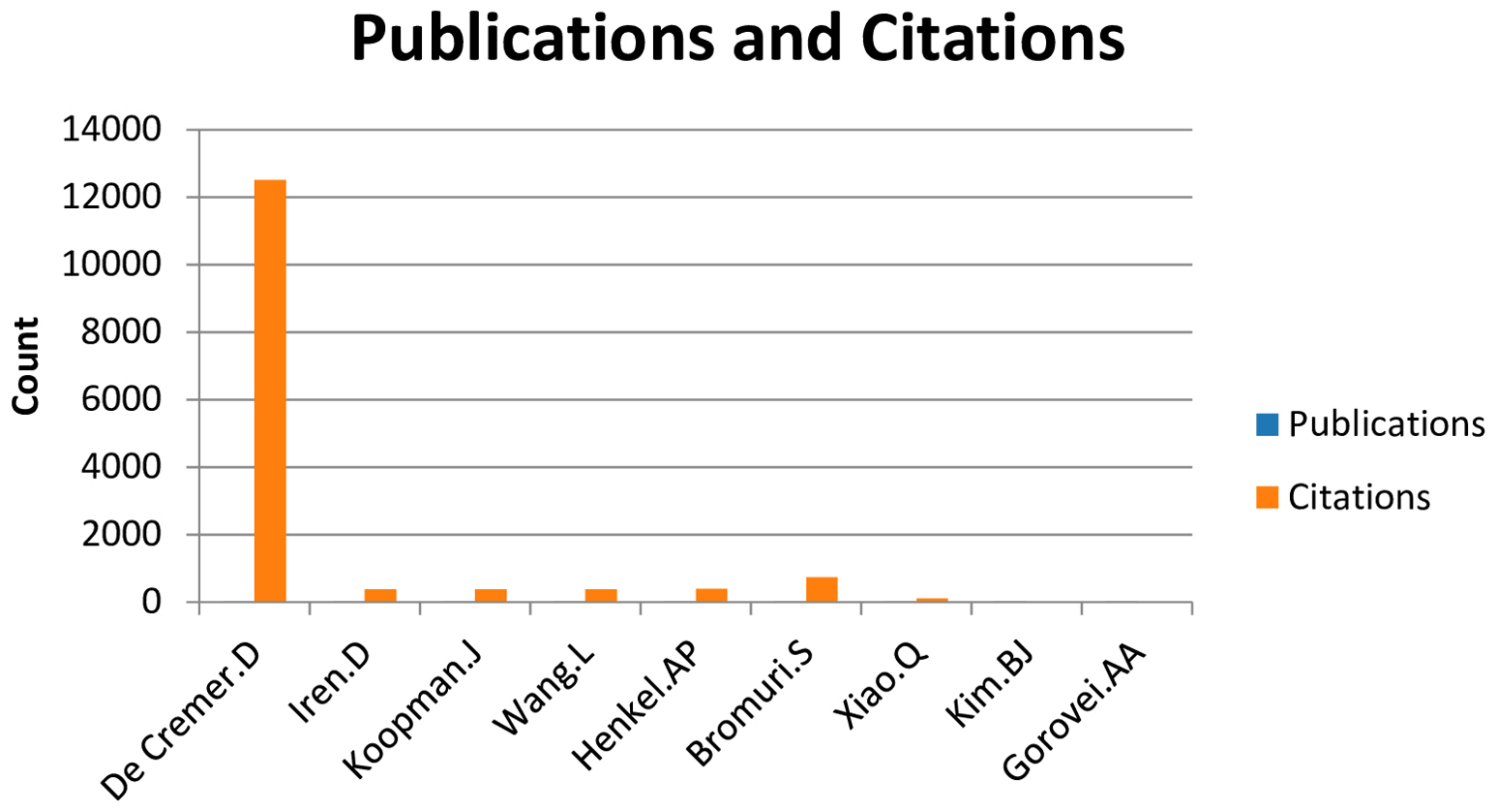
Publications per authors and citations.

The third figure illustrates the contributions of the 10 most prolific authors in the field of artificial intelligence and employee well-being. Although the field is still in its early stages, with a maximum of two publications per author, the citation analysis reveals their significant influence within the scientific community. Particularly notable are De Cremer with 12,516 citations, followed by Bromuri with 740 citations, and Byung-Jik Kim with 339 citations (Figure 3).

#### 3.1.3 Publication per countries

**Figure 4:**
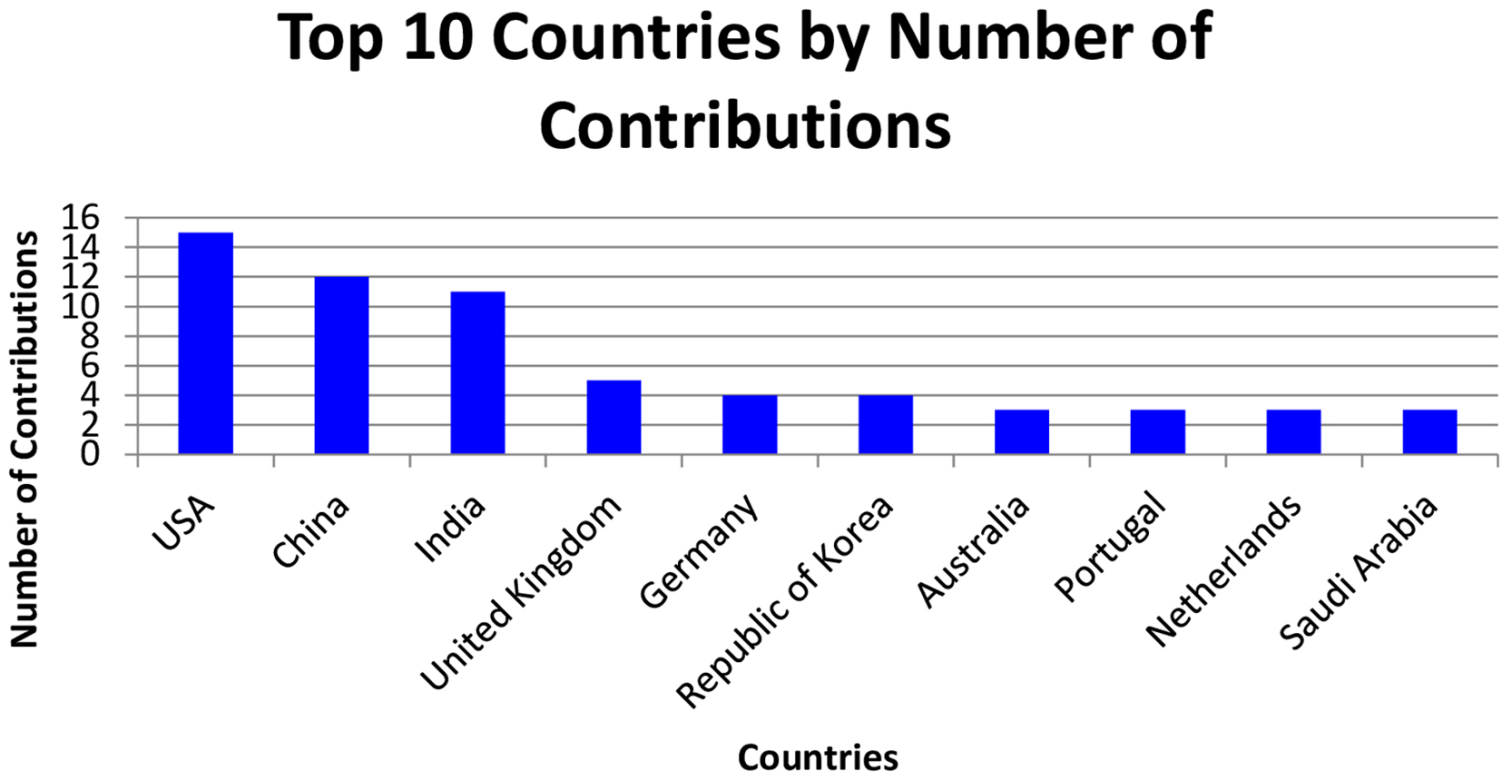
Publications per country.

The fourth figure illustrates the countries that have contributed the most to research in this field. We notice a predominance of the United States and China, followed by European countries and the United Kingdom. South Africa and Saudi Arabia are also present, and contributions from other countries such as Malaysia, Canada, Hungary, and Morocco are observed. This distribution shows a global interest in research and underscores the need to increase research efforts in other countries where contributions are reduced or non-significant, such as Taiwan, Belgium, Turkey, and Japan (Figure 4).

#### 3.1.4 Keywords clusters

**Figure 5:**
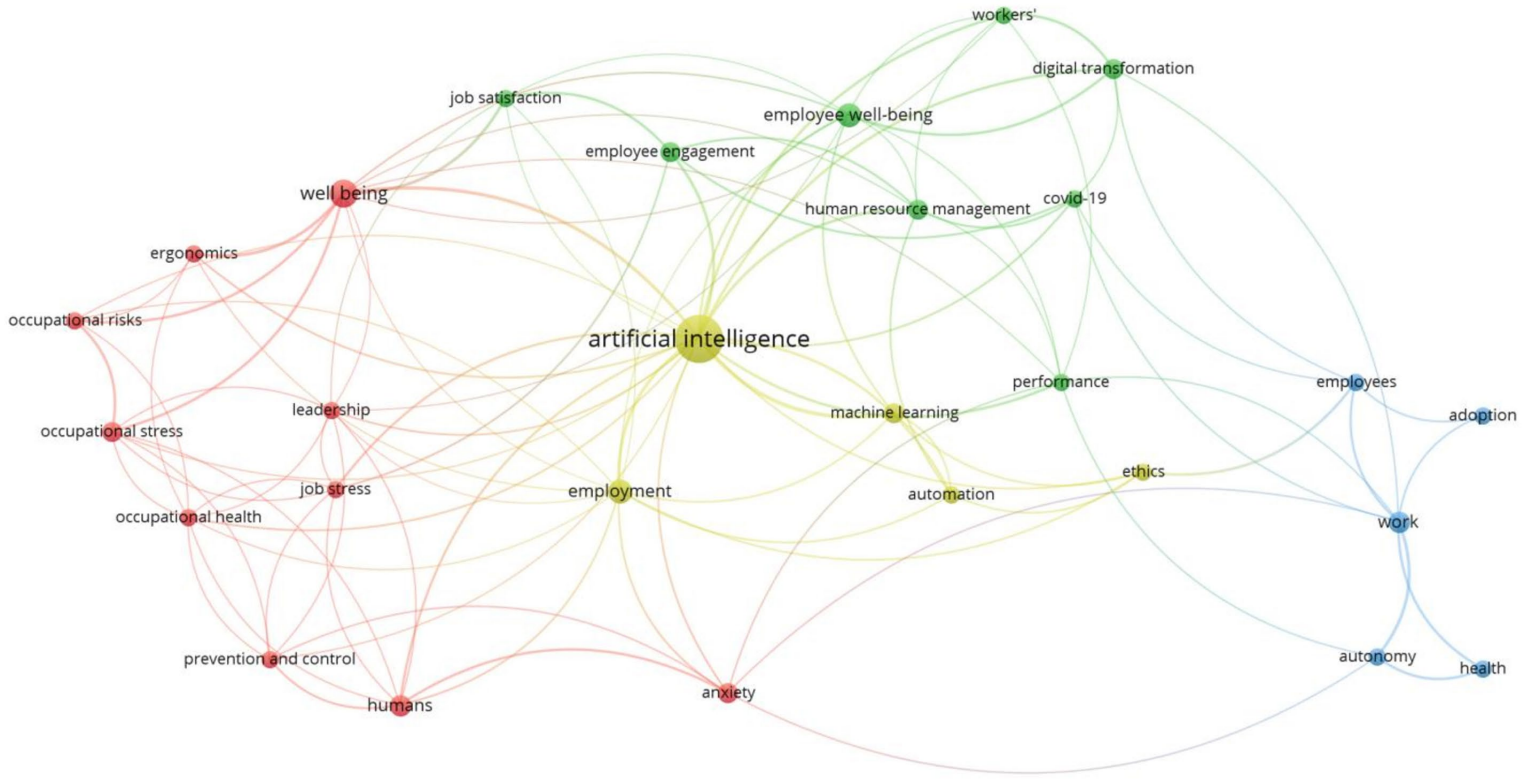
Keyword co-occurrences network.

Keywords form the foundation of published articles and illustrate the diversity of research within the studied field (Su and Lee, 2010). Through co-occurrence analysis, it is possible to identify emerging themes and analyze the interdependence between different fields of study (Chiang et al., 2023). Our keyword co-occurrence analysis has identified four main clusters that illustrate the relationship between artificial intelligence (AI) and employee well-being. Figure 5 shows the obtained visualization. The biggest number of keywords was 10, while the lowest was five (see Table 1).

**Table 1:** Keyword cluster.

Yellow	Red	Green	Blue
Artificial intelligence Employment Machine learning Automation Ethics	Anxiety Humans Job stress Occupational health Occupational stress Ergonomics Well-being	Job satisfaction Employee engagement Employee well-being Human resource management Performance COVID-19 Digital transformation Worker’s	Employees Adoption Work Autonomy Health

The yellow cluster focuses on artificial intelligence and its various forms such as machine learning and automation highlighting ethical considerations and employability. Artificial intelligence and machine learning have transformed our way of life and our way of working (Ortega-Bolaños et al., 2024) and will continue to bring significant changes in the years to come (Sanderson et al., 2023). Like any powerful technological tool the adoption of artificial intelligence within companies raises important ethical questions (Trotta et al., 2023). These ethical issues touch on various aspects such as transparency fairness data confidentiality and accountability (Ortega-Bolaños et al., 2024; Trotta et al., 2023)

Indeed, the success of an AI-based project first requires transparency; a transparent project is one that is clearly explained, understood by all stakeholders, and motivating (Morley et al., 2020). A project must also be fair, ensuring that all employees have equal opportunities and are not disadvantaged by the deployment of AI technologies (Ortega-Bolaños et al., 2024). Authors (Bollier, 2017; Gibbons, 2021) suggest that widespread internet access and the implementation of a universal basic income could help address issues related to fairness, particularly for employees who perceive the adoption of artificial intelligence in the workplace negatively. Moreover, the use and storage of data present significant ethical challenges. Every individual should have the right to manage and control their data, ensuring privacy and preventing misuse (Khan et al., 2021). Finally, accountability is a crucial pillar in the management of artificial intelligence. Accountability assigns responsibility to the company or individuals for the outcomes produced by AI systems (Benneh, 2023). In sum, these ethical considerations provide a framework for better managing the implementation of artificial intelligence in the future (Sanderson et al., 2023; Benneh, 2023).

Concerning the red cluster, it refers to the psychological and health concerns of employees in the workplace. Indeed, the emergence of artificial intelligence within companies has disrupted the future of work (Fisher et al., 2023). While AI can offer countless benefits to employers and employees, such as improved productivity and performance, its implementation can also have negative impacts. Employees with gaps in digital skills may find themselves at a disadvantage, creating social disparities and increasing the risk of job loss (Landsbergis et al., 2014). According to some studies, the fear of job loss and the stress related to acquiring new skills are considered a public health crisis (Case and Deaton, 2017). To address this and promote equity by creating a stress-free work environment for employees, several tools need to be put in place. These include continuous training for employees to bridge skill gaps, considering the cultural, societal, and environmental aspects by employers, and placing humans at the center of attention (Gibbons, 2021).

The green cluster highlights the role of digital transformation and COVID-19 on employee engagement, performance, and satisfaction. The digital transformation, accelerated by the COVID-19 pandemic, has revolutionized the world of work and business (Schilirò, 2021). The adoption of new technologies, such as artificial intelligence, creates job insecurity and affects employee engagement and performance (Ye et al., 2024; Hizam et al., 2023). To address this, acquiring new skills and improving existing ones among employees is a key element, enabling companies to enhance their competitiveness and equip employees with the necessary tools to support the change (Morandini et al., 2023; Li, 2022). Furthermore, artificial intelligence impacts not only performance and satisfaction but also managerial HR practices (Faye-Schjøll and Høye, 2020). The use of AI in human resources allows for anticipating employee departures and identifying factors contributing to their stress and dissatisfaction (Faye-Schjøll and Høye, 2020). AI also provides platforms that foster employee engagement and a sense of belonging. These tools create an attractive, collaborative work environment and drive business growth (Bhatia, 2018).

The blue cluster in the network visualization highlights the complex relationship between technology adoption in workplaces and its effects on employee, on autonomy and on health, revealing both opportunities and challenges. The integration of advanced technologies such as AI and automation often shifts employees from routine tasks to more complex and intellectually stimulating roles (Khogali and Mekid, 2023), which can significantly enhance job satisfaction and operational efficiency (Shwedeh et al., 2023; Shwedeh et al., 2023). However, this transition also introduces significant mental health risks, including job security stress and the pressure to quickly acquire new skills (Ghani et al., 2022) To effectively navigate these challenges, organizations must adopt a balanced approach by implementing comprehensive training programs that not only strengthen employees’ skills but also prepare them psychologically for technological changes (Morandini et al., 2023). Additionally, supporting employee well-being through accessible mental health resources, such as counseling and stress management workshops, and promoting policies that encourage work-life balance is crucial for alleviating work-related stress and preventing burnout (Bello et al., 2024). Furthermore, maintaining ethical standards in deploying new technologies by ensuring transparency, fairness, and data confidentiality is essential to build trust and foster an inclusive and supportive work environment (Potter and Doris, 2024). Collectively, these strategies can help organizations leverage technological advancements to improve productivity while simultaneously ensuring that employee autonomy and health are protected and promoted.

### 3.2 Content analysis

#### 3.2.1 Word cloud and clusters of the most frequently used words

**Figure 6:**
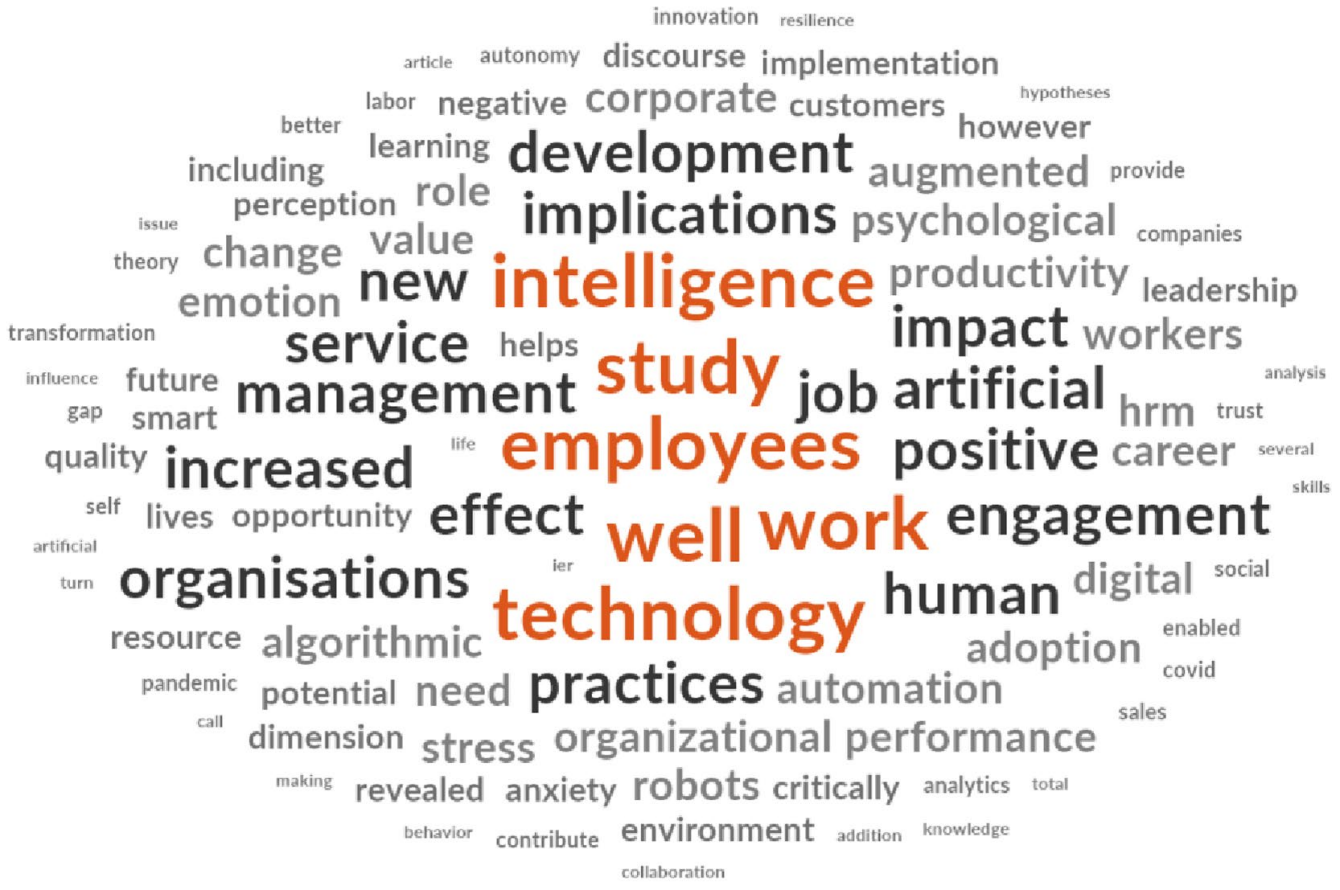
Word Cloud. Source: Output of Nvivo 14 Software.

To conduct the word frequency analysis, we used Nvivo software. The full text of the 25 selected articles was imported into the software, and the abstracts and keywords were manually encoded into a node titled “Abstracts and Keywords.” A word frequency query was then executed, and the “word cloud” option was selected to generate a graphical representation of the 100 most frequently used words in the articles. Figure 6 illustrates the most commonly used words in the abstracts and keywords of the 25 selected articles. Words appearing in larger, bold fonts, such as “employee,” were used more frequently compared to smaller words. The term “employee” stands out as a key word in the analysis. Employees are the primary actors in the digital transformation of companies. Their level of motivation, acceptance of change, and engagement are critical factors that can significantly influence the success of a company’s digital transition (Ye et al., 2024).

**Figure 7:**
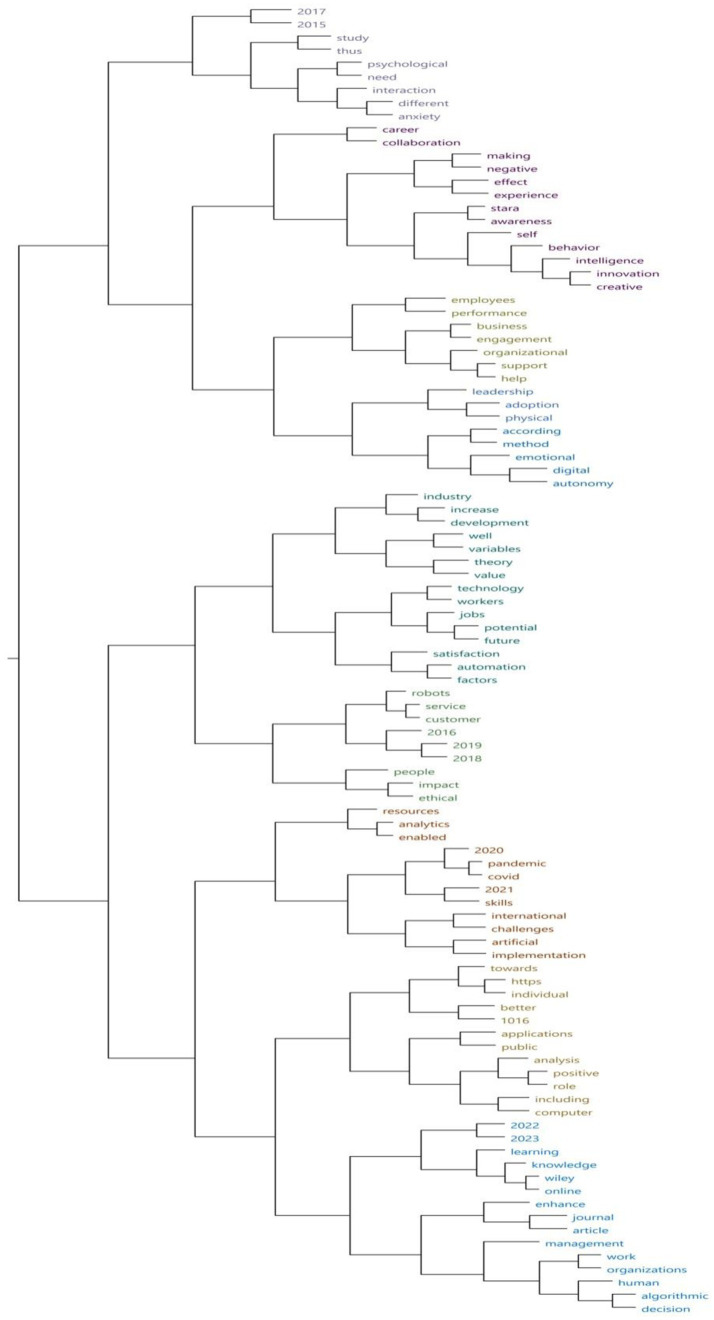
Grappes of the most frequently used words. Source: Output of Nvivo 14 Software.

To create the word cluster analysis, we also used Nvivo software. The full text of the 25 selected articles was integrated, excluding the abstracts and keywords. We then ran a “Word Frequency” query, with the results visualized as clusters to display the connections between frequently occurring words in the articles. Figure 7 illustrates the results, highlighting key associations between specific terms. For instance, the words “psychological” and “need” appeared together in a sentence, as did “career” and “collaboration.” Other notable associations include “experience” with “negative,” “employee” with “performance,” “business” with “engagement,” and “ethical” with “impact.” The pair “innovation” and “creative” is also of interest, appearing after “intelligence” and before “behavior.” Furthermore, the terms “support” and “help” often appeared together, preceded by “organizational,” while “automation” and “factors” were frequently preceded by “satisfaction.”

#### 3.2.2 The methodological approaches used by the authors

**Figure 8:**
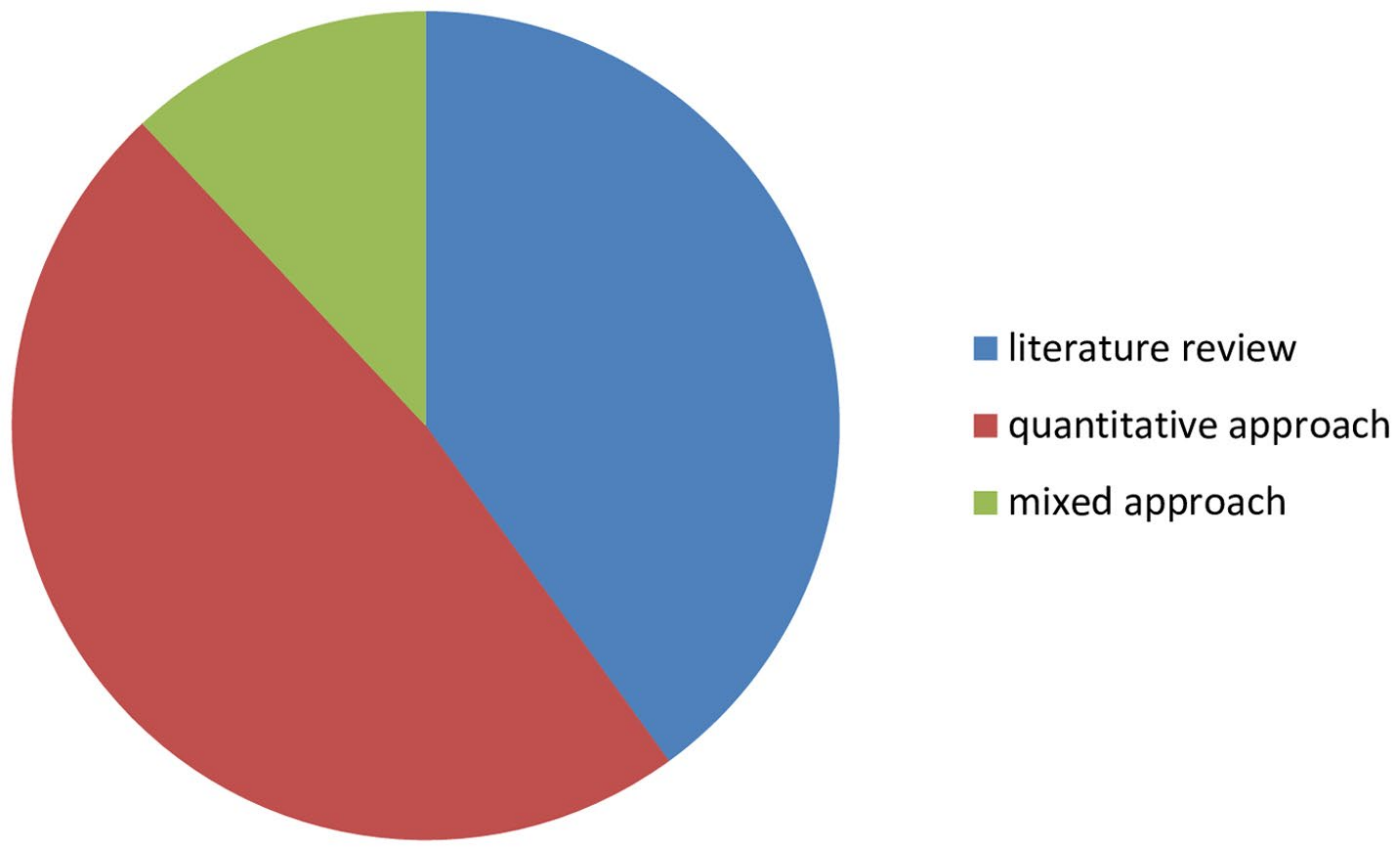
Distribution of papers by methodological approach.

In order to identify the methodological approaches used in the articles, we first conducted an in-depth reading of the papers, followed by manually categorizing the articles in an Excel table according to the approaches employed (see Figure 8). This analysis allowed us to identify three main methodological approaches adopted by the authors. First, literature reviews in various forms were undertaken by 10 articles: narrative review (Khogali and Mekid, 2023; Alavi and Habel, 2021; Bankins, 2021; Chang, 2020), systematic review (Cramarenco et al., 2023; Mer and Virdi, 2023; Mera and Srivastavab, 2023; Zhang et al., 2022) and descriptive and exploratory literature review (Gorovei, 2020). Second, the quantitative approach was employed in 12 articles. For instance, some authors (Stamate et al., 2021; Jeong et al., 2024; Jiang et al., 2022; Kinowska and Sienkiewicz, 2022; Yin et al., 2024) conducted research using Structural Equation Models (SEM), while others (Nazareno and Schiff, 2021; Henkel et al., 2020; Kong et al., 2023; Xiao et al., 2023) performed regression analyses. Lastly, the mixed-methods approach was adopted by 3 articles. For example (Brougham and Haar, 2018) analyzed an open-ended question within a quantitative questionnaire, while (Konuk et al., 2023; Correia Loureiro et al., 2023) conducted semi-structured interviews followed by a quantitative study.

#### 3.2.3 Theoretical frameworks mobilized by authors

To create the table presenting the theories utilized in the various articles, we thoroughly read all 15 articles (from the 25 initially selected, we excluded 10 that conducted literature reviews and kept only 15 that carried out empirical studies, whether qualitative, quantitative, or mixed). We manually filled out the table, counting the number of articles in which each theory was applied, and specifying the authors of those articles. We identified 20 theoretical frameworks used across these articles, which we synthesized in Table 2. Interestingly, only the first four theoretical frameworks were discussed in two or more articles, while all other theories were employed by different authors without repetition across other studies.

**Table 2:** Theoretical frameworks mobilized in the papers.

Theory used	Authors
Job demands-resources (JDR) model	Chang (2020) Xiao et al. (2023) Stamate et al. (2021)
Transactional model of stress (TMS)	Xu et al. (2023) Yin et al. (2024)
Self-determination theory (SDT)	Kinowska and Sienkiewicz (2022) Stamate et al. (2021)
Conservation of resources (COR) theory	Jeong et al. (2024) Xu et al. (2023)
Job replacement model	Chang (2020)
Job Characteristics theory	Gorovei (2020)
Psychological contract model	Chang (2020)
Biopsychosocial model	Jeong et al. (2024)
Technostress theory	Jeong et al. (2024)
Stress and coping theory	Correia Loureiro et al. (2023)
Social affiliation theory	Tang et al. (2023)
Social cognitive theory	Shaikh et al. (2023)
Theory on attachment styles/attachment theory	Tang et al. (2023)
Career-planning model	Brougham and Haar (2018)
SOR (Stimuli organism response) model	Jiang et al. (2022)
Process-focused HRM perspective/HR process approach	Xiao et al. (2023)
HRM system strength theory	Xiao et al. (2023)
Person–environment (P–E) fit theory	Kong et al. (2023)
Technology acceptance model (TAM) and its related theoretical models (e.g., the model for mandatory use of software technologies [MMUST])	Stamate et al. (2021)
Digital Taylorism as the modernization of Taylor’s original scientific management theory	Konuk et al. (2023)

Source: Elaborated by us.

#### 3.2.4 Business sectors covered by empirical studies

**Figure 9:**
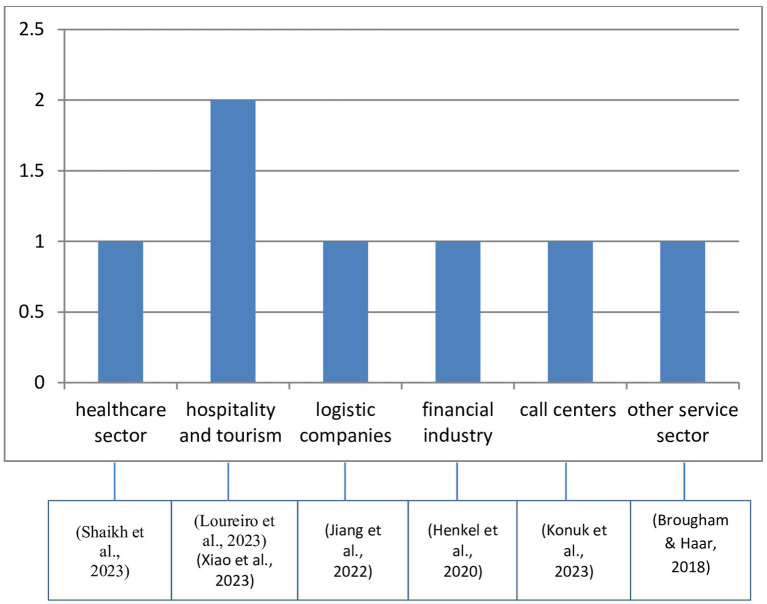
Distribution of papers with service sector empirical study.

Similarly, for sectoral analysis, we reviewed the 15 empirical studies and created a table showing the percentage of studies focused on various sectors. Of these, 8 conducted research in varied sectors, while 7 targeted specific ones. A bar chart was created to represent the distribution of sectors across the 7 targeted studies (see Figure 9).

#### 3.2.5 Topics covered and main results

In this section, we will outline the primary themes explored and the key findings reported by the authors through narrative descriptions. It’s noteworthy that while a few articles may be categorized thematically in pairs or small groups, the majority of articles introduce unique themes not revisited in subsequent works. This characteristic underscores the subject’s recent emergence and the field’s wide-open terrain, ripe for further investigations aimed at validating or challenging earlier findings. With the exception of a few unique cases, the analyzed articles can be categorized into five thematic groups. Firstly, a set of articles focuses on employees’ attitudes and perceptions regarding the opportunities and threats posed by AI, and how these perceptions impact their workplace well-being. Regarding employees’ attitudes and perceptions of the opportunities and threats posed by AI, the findings are not homogeneous. Some studies highlight that general skepticism toward job displacement from automation is prevalent, with greater awareness of STARA (Smart Technology, Artificial Intelligence, Robotics, and Automation) correlating with reduced organizational commitment and career satisfaction, as well as higher turnover intentions, depression, and cynicism among employees (Brougham and Haar, 2018). Other findings indicate that while job-replacement anxiety does not significantly impact psychological well-being (PWB), AI learning anxiety is associated with decreased PWB (Konuk et al., 2023). Conversely, some results show a positive link between the perception of AI opportunities and workplace well-being (WWB), partially mediated by informal learning in the workplace (ILW), with unemployment risk perception (URP) moderating this relationship (Xu et al., 2023). Trust in AI enhances both employee well-being and supervisor-rated productivity through improved employee-AI collaboration, particularly among those with high protean career orientations (Kong et al., 2023). Overall, general mental ability (GMA) shapes perceptions of AI’s usefulness, which affects attitudes toward the technology and ultimately influences employee satisfaction, the fulfillment of basic psychological needs, and overall psychological health (Stamate et al., 2021). Secondly, another group addresses the effects of AI and automated technologies on employee well-being, particularly emphasizing job stress and its negative impact on physical and mental health, as well as employees’ behavioral responses to this stress. Thus, the adoption of automated technologies at work consistently affects worker well-being in terms of job stress and overall health, with these effects being more pronounced in jobs at higher risk of automation (Nazareno and Schiff, 2021). Also, there is a strong association between AI adoption, heightened job stress, negative impacts on physical health, and the significant moderating effect of coaching leadership in alleviating AI-related stress (Jeong et al., 2024). Benign stress from AI adoption does not directly affect employee happiness but does so indirectly through employee engagement, which positions engagement as a key mediator between AI adoption and overall well-being (Correia Loureiro et al., 2023). Furthermore, employees with higher anxious attachment are more sensitive to AI interaction and may react by maladapting behavior by isolating themselves, experiencing insomnia or increased alcohol consumption due to their need for affiliation and feelings of loneliness (Tang et al., 2023). Providing service employees with AI tools for emotion recognition may initially raise stress levels, but also benefit well-being by raising employees’ sense of goal achievement through enhancing their ability to manage customer emotions (Henkel et al., 2020). A third group examines the contentious impact of AI adoption on job autonomy and its subsequent effects on employee well-being. The findings indicate that AI adoption promotes workplace flexibility, employee confidence, and job autonomy, all of which positively contribute to Employee Mental Health and Well-Being (EMHWB) (Shaikh et al., 2023). Conversely, the application of automated and algorithmic management practices significantly affects job autonomy, leading to indirect and potentially undesirable effects on well-being (Kinowska and Sienkiewicz, 2022). Some other articles investigate the positive influence of AI adoption on employee well-being through enhanced self-efficacy. Smart technology plays a crucial role in enhancing employee self-efficacy, which in turn has a positive influence on their overall well-being (Jiang et al., 2022). AI assistants with high intelligence not only indirectly boost employees’ AI-enabled innovation behaviors by enhancing creative self-efficacy—an effect that is amplified in organizations with greater AI readiness—but they can also have a negative impact on these behaviors through increased awareness of Smart Technology, Artificial Intelligence, Robotics, and Algorithms (STARA), particularly when organizational readiness for AI is lower (Yin et al., 2024). Lastly, a final group explores how AI contributes to tailoring workstations to meet employees’ needs, thereby improving their well-being. The evolution of the Internet-of-Things (IoT) and artificial intelligence has significantly contributed to the development of smart offices capable of understanding employees’ contexts and adapting to their needs (Zhang et al., 2022). Furthermore, job crafting mediates the positive impact of AI-enabled HR analytics on enhancing employee resilience, which, as we know, leads to employee well-being, while there is a positive moderating effect of HRM system strength on the link between AI-enabled HR analytics and job crafting (Xiao et al., 2023). The results discussed above primarily focus on the impact of AI use on employee well-being, as this constitutes the central research question of the paper. However, it is worth extending this inquiry to explore the potential effects of AI on employee productivity and performance. Consequently, in the following paragraph, we will briefly outline the key findings from the articles analyzed that address these additional dimensions, providing a more comprehensive understanding of AI’s broader organizational implications. Indeed, the question of how AI affects employee performance or productivity adds another important dimension to the discussion of well-being. For example, AI adoption promotes workplace flexibility, employee trust and autonomy, all of which contribute positively to EMHWB and, consequently, employee productivity (Shaikh et al., 2023). In addition, trust in AI positively influences supervisor-assessed productivity through improved collaboration between employees and AI (Kong et al., 2023). In terms of employee performance, smart technologies have a significant impact on company trust, self-efficacy, employee well-being and learning performance (Jiang et al., 2022). At the same time, the application of automated and algorithmic management practices significantly affects professional autonomy, with indirect and potentially undesirable effects on well-being and performance (Kinowska and Sienkiewicz, 2022).

#### 3.2.6 Research perspectives

**Figure 10:**
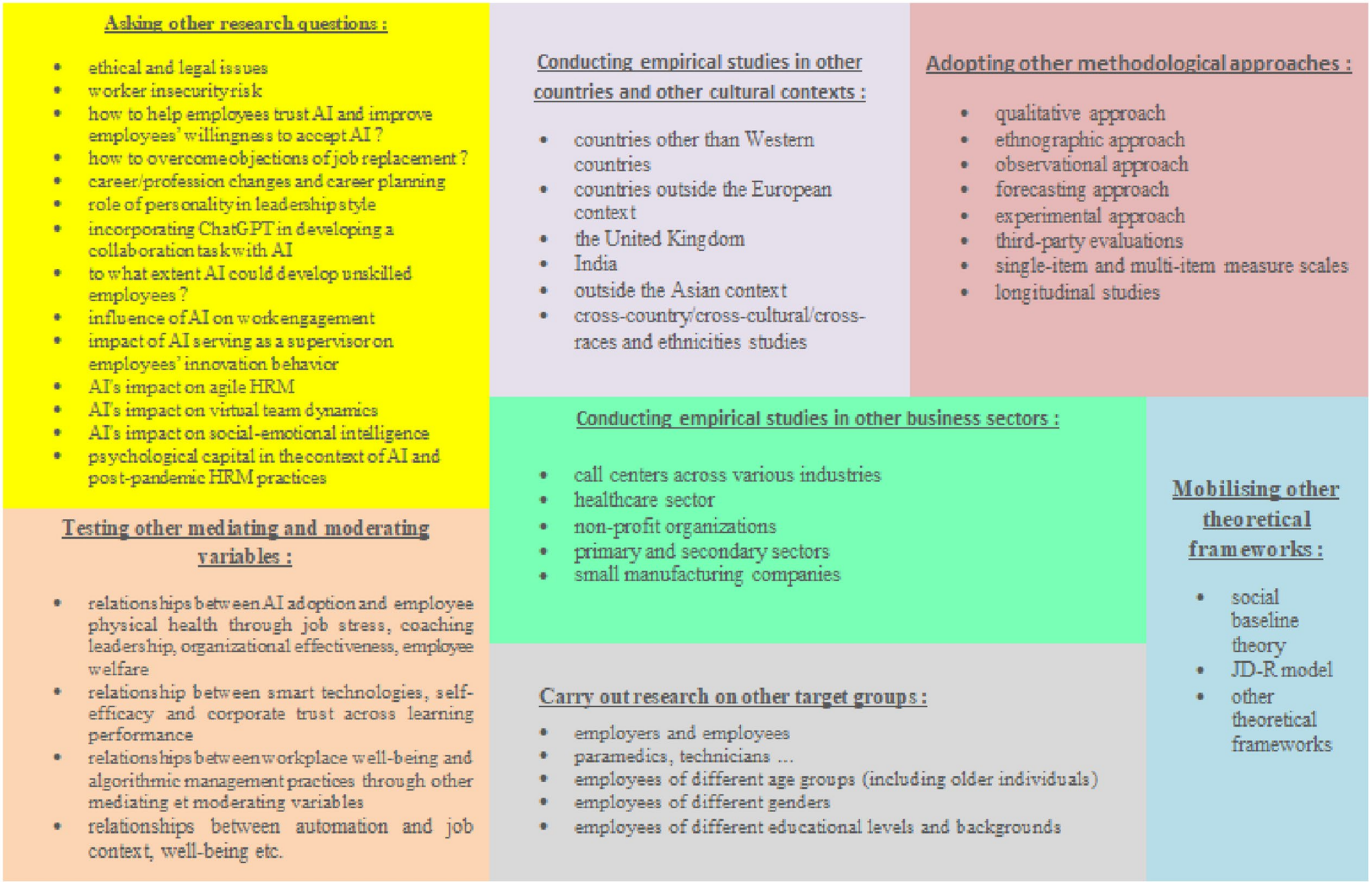
Hierarchical diagram of research perspectives based on thematic analysis. Source: Output of Nvivo 14 Software revised by us.

Through an in-depth analysis of existing literature and using the Nvivo14 software, we have identified several research perspectives that can provide avenues for future studies on employee well-being and artificial intelligence. Figure 10 depicts a hierarchical diagram illustrating research perspectives identified through thematic analysis conducted with Nvivo software, we examined the “discussion of results and research perspectives” sections from all 25 articles. Major research perspectives were encoded into nodes (e.g., “asking other research questions” “adopting other methodological approaches“). Within each node, sub-nodes were created to capture more detailed suggestions. Using Nvivo’s ability to count and visualize references (articles encoded in each node), we identified similarities across the articles in terms of suggested research perspectives. Several articles mentioned the same research perspectives, such as the need for empirical studies in other countries (8 articles) or studies targeting other groups (7 articles). Once the encoding was complete, we performed a “Hierarchical Diagram” query, which visually mapped the distribution of research perspectives across the articles. The size of each rectangle in the diagram represented both the number of articles addressing a particular perspective and the volume of text devoted to it. Larger rectangles indicated greater emphasis on those perspectives. For instance, the need for posing additional research questions emerged as a key focus, as seen by the prominence of its corresponding rectangle in the diagram. This visual representation allowed for an intuitive understanding of which research perspectives were prioritized across the literature. While Nvivo does offer the ability to generate diagrams that include both nodes and sub-nodes, the result was overly complex and hard to interpret. To address this, we refined the diagram manually using Word, applying graphic elements to incorporate key sub-nodes (or sub-themes) into the corresponding rectangles. This approach helped to clarify, for example, which additional research questions were proposed by the authors or which new target groups were suggested for future empirical studies. The goal was to create a clear, concise visual summary, allowing readers to quickly grasp the main points before engaging with the detailed text that followed. In the upcoming text, we will elaborate on the various research perspectives referenced by the authors of the 25 selected articles.

##### 3.2.6.1 Testing other moderating and mediating variables

Most quantitative empirical studies suggest further research to test additional mediating and moderating variables within their proposed conceptual models, such as job crafting, cognitive reappraisal, self-esteem (Xu et al., 2023) or psychosomatic well-being, cognitive and professional elements (Cramarenco et al., 2023).

##### 3.2.6.2 Conducting empirical studies in other countries and other cultural contexts

Indeed, there is a need for more comprehensive research to encompass other countries, especially beyond the European context (Stamate et al., 2021; Henkel et al., 2020). Similarly, it is important to conduct studies in cultural settings beyond China and other Asian countries, particularly focusing on regions such as the United Kingdom and India (Kong et al., 2023). Furthermore, there is a necessity for cross-country and cross-cultural studies (Kong et al., 2023; Correia Loureiro et al., 2023), as well as research examining different races and ethnicities (Nazareno and Schiff, 2021).

##### 3.2.6.3 Mobilizing other theories and theoretical frameworks

From the perspective of social baseline theory, interactions with AI in the workplace may be seen as a form of “relationship disruption” which can trigger a social-regulatory process marked by heightened uncertainty and perceived risk, leading individuals to actively work toward preserving social relationships (Tang et al., 2023). Future research on the impact of AI on work engagement, as well as the investigation of the dual effects of AI-assisted intelligence on employees’ innovation behaviors, could benefit from using the JD-R model as a foundational framework (Mera and Srivastavab, 2023); (Yin et al., 2024). Additionally, future studies might gain from integrating additional theoretical frameworks that account for the myriad factors influencing employee productivity, such as organizational culture and leadership styles (Shaikh et al., 2023).

##### 3.2.6.4 Carry out research on other target groups

Research on workplace well-being should integrate perspectives from both employers and employees, as well as other relevant stakeholders (Kinowska and Sienkiewicz, 2022). Additionally, the inclusion of healthcare employees, alongside patients, is crucial to fully understanding well-being in the healthcare sector (Mera and Srivastavab, 2023). To generalize findings, it is important to expand studies beyond doctors to include paramedics, technicians, and other support staff (Shaikh et al., 2023). Given the prevailing emphasis on younger populations in existing research, it is essential to explore the perceptions of older participants and compare behaviors across various age groups (Stamate et al., 2021; Correia Loureiro et al., 2023). Considering the complex socio-technical dynamics associated with automation and AI, studies should also examine gender differences (Nazareno and Schiff, 2021). Moreover, individuals with a background in technology or engineering, along with higher levels of knowledge and skills, may experience enhanced collaboration with AI (Ahn et al., 2022; Oksanen et al., 2020). Therefore, it is vital to diversify research samples to include individuals with lower educational backgrounds, moving beyond the predominant focus on well-educated groups (Yin et al., 2024; Xu et al., 2023).

##### 3.2.6.5 Adopting other methodological approaches

Comprehensive research employing a diverse range of qualitative and quantitative methods—such ethnographic, survey, observational, forecasting, and experimental approaches—is essential (Nazareno and Schiff, 2021). Furthermore, to mitigate common method bias and enhance reliability, researchers should incorporate third-party evaluations alongside self-report measures (Xu et al., 2023). Moreover, to reduce response biases, it is crucial to utilize robust measurement techniques with higher content validity, including multi-item scales in addition to single-item measures (Stamate et al., 2021). Longitudinal studies are also necessary to capture changes over time (Henkel et al., 2020; Xiao et al., 2023; Brougham and Haar, 2018; Konuk et al., 2023).

##### 3.2.6.6 Conducting empirical studies in other business sectors

Studying the effects of AI in various industries and service settings is crucial (Nazareno and Schiff, 2021). The healthcare sector emerges as a particularly promising area for AI adoption, especially considering the vulnerabilities highlighted during the COVID-19 pandemic (Mera and Srivastavab, 2023). Comparative analyses between public and private hospitals could deepen our understanding of AI’s role in healthcare (Shaikh et al., 2023). Additionally, research in call centers, including non-profit organizations like municipalities, is vital due to differing operational dynamics (Konuk et al., 2023). Future studies should also include employees from both primary and secondary sectors (Brougham and Haar, 2018). Lastly, examining small manufacturing companies is essential, as workers in these settings may face unique challenges associated with AI, often stemming from lower education levels and a lack of necessary skills and resources (Xiao et al., 2023).

##### 3.2.6.7 Asking other research questions

Future research on AI-enabled HR analytics should not only focus on its positive impacts but also address potential risks and challenges, including ethical and legal issues as well as negative employee experiences (Xiao et al., 2023). Investigating ethical norms in the workplace context of AI use is essential, particularly regarding the implications of job losses and gains amid rapid technological advancements (Khogali and Mekid, 2023). The unresolved issue of worker insecurity due to automation warrants further investigation, as it could significantly affect employee well-being (Nazareno and Schiff, 2021).

Moreover, while concerns about job displacement are prevalent, AI is becoming an integral part of future workplaces. Research should emphasize educating and training employees on AI applications to build trust and alleviate fears of job loss (Correia and Matos, 2021). Strategies that enhance employee trust in AI and facilitate acceptance of its presence in the workplace could also be explored, particularly regarding how AI technologies impact work-life balance (Kong et al., 2023). Additional research questions may include examining the effects of AI on cognitively demanding jobs and identifying management strategies to empower employees in their interactions with AI rather than fostering feelings of threat (Chang, 2020). Although there is a concern about job displacement due to Smart Technology and Advanced Robotics Applications (STARA), it is likely that new job categories will emerge, necessitating studies on career planning and the potential for workforce transitions (Brougham and Haar, 2018). In the healthcare sector, effective management of AI through technological leadership remains a challenge, highlighting the need for studies on the influence of leadership personality on management styles (Shaikh et al., 2023). Scholars could also consider integrating open AI systems, such as ChatGPT, into collaborative tasks (Tang et al., 2023). Future investigations should assess how AI, automation, and digitalization can enhance the skills of unskilled workers in advanced economies (Mer and Virdi, 2023). Furthermore, exploring AI’s impact on agile HRM, virtual team dynamics, social–emotional intelligence, and psychological capital within post-pandemic HRM practices is crucial (Mer and Virdi, 2023). Finally, research should delve into AI’s role in enhancing work engagement (Mera and Srivastavab, 2023) and examine the effects of AI as a supervisory entity on employees’ innovation behaviors (Yin et al., 2024).

## 4 Discussion

This article investigates how integrating artificial intelligence (AI) in businesses affects employee well-being. The integration of AI into professional environments has both positive and negative effects on employees as well as on the future of employment (Mera and Srivastavab, 2023). Our study has shown that the positive impacts of AI include the automation of repetitive and stressful tasks, along with improvements in flexibility, confidence, autonomy, and collaboration among employees (Shaikh et al., 2023). However, some employees view these changes negatively, fearing potential job loss and mental health issues, such as depression (Brougham and Haar, 2018).

To ensure successful AI integration, it is crucial to address several aspects. The ethical dimension is vital, encompassing transparency, fairness, data confidentiality, and accountability (Khogali and Mekid, 2023; Bankins, 2021). Moreover, ongoing training and coaching are necessary to reduce stress and alleviate fears of job loss among employees (Bello et al., 2024). These measures will help mitigate the negative effects of AI and enhance its management within the company.

Our systematic analysis has revealed several gaps in the current literature, which has led us to suggest new avenues for future research. We have identified the need to broaden the geographic scope of AI studies beyond the United States, Asia, and Europe, with a particular focus on less-researched sectors such as education and call centers. To gain a deeper understanding of AI’s impact on employee well-being, our findings indicate the necessity of employing diverse methodologies, including qualitative approaches and longitudinal studies. Our analysis also highlights the importance of exploring how various demographic groups, taking into account age, sex, gender, and culture, perceive the adoption of AI. It is crucial to pay special attention to the ethical implications of AI in professional environments, issues related to job security, and the significant role that training can play in boosting employees’ confidence and adaptability to AI. Additionally, our study suggests that an examination of leadership and management strategies in AI integration could yield important insights. These perspectives are directly derived from our analysis, underscoring a critical need to address these identified gaps in future research.

To conclude, while our study initially aimed to analyze the relationship between artificial intelligence (AI) and employee well-being, our review of the literature revealed that the impact of AI extends beyond well-being to encompass employee engagement, productivity, performance, and satisfaction (Tang et al., 2023).

## 5 Conclusion

This article has explored the complex interplay between artificial intelligence (AI) and employee well-being through a detailed bibliometric review and contextual analysis. Our findings highlight the dual nature of AI’s impact: enhancing efficiency and engagement on one hand, while potentially increasing job stress and insecurity on the other (Morandini et al., 2023). Significant gaps were identified in the existing literature, particularly concerning the sectors studied, methodologies applied, and theories employed. The adoption of AI in professional settings presents substantial ethical and organizational challenges that necessitate careful management to maximize benefits while minimizing risks to employee well-being (Olatoye et al., 2024).

These results corroborate the findings of Stamate et al. (2021), who suggest that employees’ attitudes toward AI significantly influence their job satisfaction and psychological well-being. Moreover Calvo et al. (2020) and (Nazareno and Schiff, 2021) indicates that while AI can offer opportunities, it also poses potential threats of instability and stress for workers undergoing rapid technological transitions.

Future research in this domain is essential to develop a nuanced understanding of AI’s interaction with employee well-being. This necessity for further investigation is underscored by Ortega-Bolaños et al. (2024), who call for a deeper exploration of the ethical issues associated with AI in the workplace. Additionally, Fisher et al. (2023) emphasize the need to examine AI’s impacts on occupational health and safety equity. Indeed, good mental health among employees can significantly enhance an organization’s competitiveness. An organization whose employees suffer from mental health issues may face detrimental impacts on its financial and operational health. This can lead to problems such as decreased productivity and concentration, as well as an increase in absenteeism and turnover rates (Dimoff et al., 2014; Dimoff and Kelloway, 2018). To mitigate these risks, organizations looking to maintain their performance and the well-being of their employees should consider implementing artificial intelligence solutions that address the psychological needs of employees. It is crucial to use AI cautiously, tailoring its application to the specific needs, age, and educational level of each employee (Wei and Li, 2022).

Future research should also incorporate the theoretical perspectives mentioned, particularly those concerning data privacy and transparency in AI processing. Additionally, as emphasized by Gibbons (2021), ongoing education for employees on AI technologies and support for their adaptation are essential to minimize potential negative impacts and ensure a technological integration that improves workplace well-being rather than detracting from it. The findings of our study contribute to the existing literature on artificial intelligence (AI) and employee well-being at three levels: theoretical, practical, and methodological. Theoretically, this research paves the way for new perspectives by analyzing the intersection of AI and employee well-being through theoretical frameworks such as the “Social Baseline Theory” and the “JD-R Model.” The results have also highlighted the need to explore these themes in various research contexts, including in small manufacturing companies, call centers, and the non-profit sector, as well as through diverse targets such as comparative studies between employees and employers, between different age groups, and those with lower education levels. Practically, although our study is theoretical in nature, it has revealed key points that allow organizations to fully benefit from the potential of AI to improve performance and employee well-being. Indeed, successful adoption of AI requires organizations to implement certain ethical standards such as transparency and fairness, as well as effective AI management based on ongoing training of employees to enhance their skills, placing them at the center of attention to preserve their well-being and mental health. Methodologically, our study has developed a solid theoretical framework for studying artificial intelligence and well-being, allowing future researchers to further explore this field. We have also identified several methodological approaches to adopt in future research, such as ethnographic or longitudinal studies, to assess long-term impacts. In summary, in an era marked by rapid digital transformation, successful adoption of AI requires an approach that promotes the integration of all stakeholders and relies on close collaboration between them, in order to develop strategies that combine employee well-being and organizational performance (Dikshit et al., 2024).

## Data Availability

The raw data supporting the conclusions of this article will be made available by the authors, without undue reservation.
